# Training-Induced Functional Gains following SCI

**DOI:** 10.1155/2016/4307694

**Published:** 2016-06-15

**Authors:** P. J. Ward, A. N. Herrity, S. J. Harkema, C. H. Hubscher

**Affiliations:** ^1^Department of Anatomical Sciences & Neurobiology, University of Louisville, Louisville, KY 40202, USA; ^2^Kentucky Spinal Cord Injury Research Center, University of Louisville, Louisville, KY 40202, USA; ^3^Frazier Rehab Institute, University of Louisville, Louisville, KY 40202, USA; ^4^Department of Neurological Surgery, University of Louisville, Louisville, KY 40202, USA

## Abstract

We previously demonstrated that daily, hour-long training sessions significantly improved both locomotor (limb kinematics, gait, and hindlimb flexor-extensor bursting patterns) and nonlocomotor (bladder function and at-level mechanical allodynia) functions following a moderate contusive spinal cord injury. The amount of training needed to achieve this recovery is unknown. Furthermore, whether this recovery is induced primarily by neuronal activity below the lesion or other aspects related to general exercise is unclear. Therefore, the current study objectives were to (1) test the efficacy of 30 minutes of step training for recovery following a clinically relevant contusion injury in male Wistar rats and (2) test the efficacy of training without hindlimb engagement. The results indicate that as little as 30 minutes of step training six days per week enhances overground locomotion in male rats with contusive spinal cord injury but does not alter allodynia or bladder function. Thirty minutes of forelimb-only exercise did not alter locomotion, allodynia, or bladder function, and neither training protocol altered the amount of in-cage activity. Taken together, locomotor improvements were facilitated by hindlimb step training for 30 minutes, but longer durations of training are required to affect nonlocomotor systems.

## 1. Introduction

From lower vertebrates to humans, it is known that locomotor activity is generated by spinal neuronal circuits referred to as the central pattern generator (CPG) [1]. Studies in lower vertebrates have been useful for modeling how the CPG performs in the absence of supraspinal and afferent feedback as well as determining the roles of neurotransmitters and afferent stimuli (load, speed, and perturbations). Within the context of spinal cord injury (SCI), central pattern generation has become a conceptual basis for locomotor training after injury [2, 3].

In rats, cats, and humans, a large amount of spontaneous recovery can occur following SCI, and this recovery is closely related to white matter sparing [4–6]. Specifically, this recovery has mainly been attributed to spared fibers in the ventral and ventral lateral funiculi where the rubrospinal, reticulospinal, and vestibulospinal tracts are located [7, 8]. Yet, the recovery achieved via training is generally specific to the task practiced, for example, stand, step, or swim training [9, 10]. The amount of activity imposed on the limbs is also a crucial component of locomotor rehabilitation. Importantly, step training on a treadmill using body weight support should provide a high number of repetitions to facilitate motor learning [11]. Recovery potential therefore is a function of (1) amount of sparing/injury severity, (2) task specificity, and (3) amount of activity (activity dependent plasticity). Other aspects of step training or general exercise may also contribute to SCI recovery, including environmental enrichment, intermittent hypoxia, and general improvements in body strength and psychological well-being. More studies are needed to determine the efficacy and mechanisms of training on functional outcomes (including locomotion, allodynia, and autonomic functions) after incomplete contusions in combination with appropriate control groups, kinematics, and overground locomotion (for review see [12]).

In humans, complete cord transection is rare. Therefore, a clinically relevant rat contusion model of SCI may provide very useful information for the study and translation of locomotor training. The rat model exhibits similarities to human SCI progression [13]. Research with spinally complete and incomplete rodents has affirmed central pattern generation and identified important aspects of training and SCI locomotion [11, 14, 15]. While it is clear that training influences aspects of treadmill locomotion post-SCI, basic research studies have yielded conflicting outcomes regarding overground locomotion [16–19]. Furthermore, very little is known about the efficacy of step training on nonlocomotor functions.

We previously demonstrated that 60 minutes of training 7 days per week significantly improved both locomotor and nonlocomotor functions, such as open field locomotion, hindlimb kinematics, at-level mechanical allodynia, and bladder function in contused rats [20]. In the current study, we (1) tested the efficacy of a 30-minute body weight supported treadmill step training paradigm for the recovery of overground locomotion using qualitative scoring (BBB) and quantitative (kinematic) locomotor tests, (2) determined the contribution of in-cage activity to spontaneous locomotor recovery versus training-induced recovery, and (3) examined bladder function and allodynia (pain response to a nonnoxious stimulus). SCI control groups (nontrain, forelimb) were identically handled, harnessed, and tested. The SCI forelimb trained control group addressed the potential for exercise mediated improvements. Sham (laminectomy only) animals provided a standard comparison throughout the study for each parameter.

## 2. Materials and Methods

All animal procedures were performed according to the NIH guidelines, and the protocols were reviewed and approved by the Institutional Animal Use and Care Committee at the University of Louisville, School of Medicine. Thirty-eight male Wistar rats (Harlan Sprague Dawley, Inc., Indianapolis, IN) approximately 60 to 70 days old weighing approximately 300 grams were individually housed in an animal room with a 12-hour light and dark cycle. Four animals served as shams (laminectomy controls). SCI animals were randomly divided into three groups before training began as previously described [20]. One group (*n* = 14) was quadrupedally trained; a second group (*n* = 10) served as nontrained controls; a third group was forelimb trained (*n* = 10). Training began at two weeks after SCI for thirty minutes per day, six days per week and continued for six weeks.

### 2.1. Spinal Cord Injuries

Animals were anesthetized with an intraperitoneal dose of ketamine (80 mg/kg, Ketoset®, Fort Dodge Laboratories, Fort Dodge, IA) and xylazine (10 mg/kg, AnaSed, Lloyd Laboratories, Shenandoah, IA) mixture. Chlorhexiderm scrub cleaned the shaved surgical site. Lubrication was applied to the eyes. The following were administered subcutaneously: 0.5 mL of dual penicillin (PenJect®; The Butler Company; Columbus, OH) single dose perioperatively as a general prophylactic, 5 mg/kg gentamicin (GentaFuse®, Butler Schein, Dublin, OH) once per day for 5 days to prevent bladder infections, 2.5 mg/kg ketoprofen (Ketofen®, Fort Dodge Laboratories, Fort Dodge, IA) twice per day for two days to alleviate postsurgical pain, and 10 mL saline, as previously described [20].

Heating pads maintained body temperature during surgery and throughout the recovery period. The T8 lamina was removed to expose the T9 spinal cord. Spinal clamps were applied to the T7 and T9 spinous processes to stabilize the spinal column. The Infinite Horizon impactor was used to deliver a 210 kdyn impact. Laminectomy shams had the T9 cord exposed but not contused. The muscle was closed with suture, the skin closed with Michel clips, and topical antibiotic applied. Animals were single housed on a 12 : 12 light : dark cycle.

### 2.2. Behavioral Procedures

*Training Paradigm*. Training interventions may be less effective when initiated in chronic SCI subjects. However, training interventions initiated too early after SCI may be detrimental to recovery efforts by exacerbating secondary injury cascades [21]. We initiated training at two weeks after SCI after the majority of spontaneous recovery had occurred according to past experience with this injury severity (based on locomotor scores and recovery of spontaneous bladder voiding) [22]. Step training was performed on the Exer3 Treadmill system (Columbus Instruments, Columbus, OH) customized with spring scales for body weight support. The animals were harnessed with lycra vests (Robomedica, Inc., Mission Viejo, CA) and hook-and-loop material and Velcro straps.

Trained animals began quadrupedal step training 2 weeks after SCI surgery, 6 days per week, 30 minutes per day, for 6 weeks, at 22 cm/s. Trainers adjusted the body weight support as needed using manual assistance at the hip flexor region to facilitate proper plantar placement, for example, complete toe extension, no ankle rotation, and incorporation of forelimb-hindlimb coordination with minimal assistance. Rats were encouraged to step independently as they began to gain consistent stepping and more stability without collapsing and dragging their hindlimbs. It is of note that animals were quadrupedally trained, as many studies utilize an upright bipedal training and testing position. The upright position alone can facilitate stepping [23]. Forelimb animals were harnessed and walked with forelimbs on the treadmill while a custom 4 × 6 inch metal platform supported the hindlimbs just above the moving treadmill belt. Nontrained animals were harnessed while a custom platform supported all limbs. For nontrained animals, the 4 × 12 inch metal platform was beside the treadmill belt. One trained, one nontrained, and one forelimb trained animal could be harnessed simultaneously. No body weight support was provided for forelimb and nontrained animals. Sham animals were not exposed to the treadmill system and were handled four times per week (minimum).

Rats were not required to complete the 30-minute session during the first week of training if they exhibited signs of stress, that is, porphyrin staining around the eyes or nose, irregular breathing, or excessive diarrhea. All animals were able to complete the 30 minutes by 6 sessions. Animals were never stimulated to step by perineum or tail pinching. Noxious stimuli were avoided during training sessions; for example, if an animal had skin abrasion on a paw, animals ceased from training until the issue was resolved, as potentially noxious input may inhibit spinal learning [22, 24, 25].

*Locomotor Assessment*. An open field locomotor assessment, the Basso-Beattie-Bresnahan (BBB) scale, was used to evaluate hindlimb function in the rats [26]. Once per week, each animal was placed in an open field and tested for 4 minutes by the same two scorers, who were presented with the trained, forelimb, nontrained, and sham animals in random order (blinded to group). The BBB uses a 21-point scale for locomotion, which rates parameters, such as joint movements (0–8), weight support (9–13), and paw placement (14–21). Intact animals demonstrate a locomotor score of 21, whereas animals that exhibit complete paralysis of the hindlimbs are scored as 0. Baseline measurements were collected prior to SCI surgery followed by weekly testing thereafter.

*Kinematic Data Acquisition*. After the six-week training period the hindquarters were shaved and the bony landmarks on the lateral side of the left and right hindlimbs were marked with permanent marker: iliac crest, greater trochanter, lateral malleolus, and metatarsophalangeal joint (ilium, hip, ankle, and toe). The pad on the plantar surface of the paw was also marked. Each animal was then individually placed in a clear plexiglass runway (in random order). As the animal passed from one end of the tank to the other darkened side, cameras positioned on the side and underneath the tank captured the angles and footfall patterns [27]. 2D overground (unassisted) kinematics were analyzed using the MaxTraq motion analysis system (Innovision Systems, Columbiaville, MI). The iliac crest, hip, ankle (lateral malleolus), toe, and paw were digitized by a blinded observer, and the hip-ankle-toe (HAT) and iliac crest-hip-ankle (IHA) angles were marked to quantify the movement of the hindlimbs during overground stepping. Four animals (*n* = 3 forelimb; *n* = 1 nontrain) could not generate weight bearing steps (BBB = 8; 8 weeks after SCI); these animals did not participate in the RI or PSI tests.

*Horizontal Ladder Walk*. Animals were additionally tested for fine locomotor control by crossing a horizontal ladder of metal bars (Columbus Instruments, Columbus, OH) [28, 29]. Animals were tested for their ability to correctly place their hind paws while crossing the bars. Forelimb errors were not counted. Animals with more severe deficits (BBB score < 11) were not tested because their limbs drag across the rungs and have a falsely reduced error count. The animals readily crossed the runway with minimal encouragement. Two blinded raters manually counted the number of footfall errors (hind paw/limb slip or fall through the bars). After each crossing, the raters discussed and agreed on the error count. If, at the end of the crossing, the raters did not agree on error count within two errors, the trial was repeated. Footfall error is reported as the mean of three high quality passes (crossed with little or no hesitation and without changing direction; hindlimbs did not drag; raters agreed on error count).

*At-Level Allodynia*. Behavioral testing of SCI rats for sensitivity to normally innocuous stimuli (touch and gentle squeeze/pressure) was performed using our published grading scale for the scoring of pain-like behavior to trunk stimulation in the rat [30]. Detailed methods also are described in [20]. Once per week, testing sessions commenced at approximately the same time of day (9 am before the start of training). The dorsolateral trunk (T7–T9 dermatomal level) just above the T9 spinal injury level was tested bilaterally for at-level mechanical hypersensitivity to touch and gentle touch/squeeze. Two baseline measurements (at least 3 days apart) were performed before injury for all rats. Throughout the study, all allodynia testing was consistently performed by the same two experimenters, blinded to treatment groups.

At the start of testing, the top of the cage was removed and the animal was allowed to acclimate to the environment for 2 minutes. While in its cage, each animal was stroked at the dorsolateral trunk five times bilaterally with a number 5 paintbrush (1.5 × 0.5 cm bristles; average pressure, 15 g) in an alternating rostral/caudal plane [30]. An interstimulus interval of 1 min between sides was maintained. After each stroking stimulus, the presence/absence of any evoked responses that were indicative of pain was noted: (1) a freezing response (stopping of normal activity and staying still in response to the stimulus), (2) escape (any movement of the animal away from the stimulus probe), and (3) grabbing at or pushing away the stimulus probe with their forepaws. An animal must show an evoked pain-like behavioral response at least 60% of the time in a given session to be considered responsive to the testing stimulus (i.e., an animal responded to at least three of five stimuli/strokes per side) [31]. Responses to brush, if present, were further assessed for threshold values using a set of Semmes-Weinstein monofilaments (20-filament set, 15 of which are in the range of 0.008 g to 15 g; Stoelting Co., Wood Dale, IL). Animals designated as responders to brush (15 g stimulus) were then given a numeric score based on their associated responses to filament testing, receiving a minimum of 4 (4 = freeze, 5 = escape, and 6 = grab/push—as the aggressiveness of the behavioral response increases, so does the score) to a maximum of 10 (see [30] for scoring scale).

Depending on the behavioral response of the animal to brush, an initial filament stimulus was applied by pressing the tip of the filament into the dorsolateral trunk (T7–T9) until it bent. If the animal responded, a lower gram filament was applied to test the animal's sensitivity. In between filament probing, the animal was left alone for a 1 min interstimulus interval. The process was repeated until the lowest gram filament that the animal responded to 60% of the time was determined. If the animal was not responsive to the initial probing stimulus, the next greatest gram filament (and repeated if needed) was used to determine the threshold of the animal's sensitivity.

For those animals not responsive to brush stroke (i.e., evoked pain-like behavioral response to less than 60% of the stimuli—less than three of five strokes), a gentle squeeze/pressure test was conducted to determine if the animal had increased sensitivity to a stronger mechanical stimulus over a wider surface area (which also normally does not provoke avoidance behaviors and is thus considered innocuous). In this instance, the animal's skin is gently squeezed with a pair of modified Adson tissue forceps (2.0 mm wide tips), which is equivalent to the 60 g Semmes-Weinstein monofilament. Gentle squeeze/pressure was applied to the dorsolateral trunk (T7–T9) five times bilaterally, with an interstimulus squeeze interval of 1 min. As with touch-evoked agitation, any evoked pain-like responses were observed and documented (0 = no response, 1 = freeze, 2 = escape, and 3 = grab/push). After the testing session, animals were scored for their degree of at-level allodynia based on a 10-point scale, with 10 being the maximum score an animal could receive [30]. Scores from each weekly testing were documented for each animal and averaged together to obtain a mean weekly allodynia score for each group.

*Transvesical Catheter*. After the last training session and all locomotor testing, a transvesical bladder catheter was implanted under 2% isoflurane (similar to [32]). Body temperature was maintained with a circulating water-heating pad. Briefly, the bladder was exposed via a midline abdominal incision through the skin and musculature. A purse-string suture (4-0 Ethilon) was placed in the urothelium of the bladder dome. PE-60 tubing (the tip previously heated to form a collar ~2 mm from the end) was inserted through the bladder dome within the suture limits and secured. The bladder was emptied, and the tubing was passed through the subcutaneous tissue and exteriorized behind the neck [33]. After a 1.5-hour recovery period, the animal was placed in a darkened box for cystometric recordings.

*Urodynamic Analysis via Nonstop Transvesical Cystometry*. The catheter was connected to an infusion pump and pressure transducer. Normal saline was infused into the bladder at a rate of 0.25 mL/min to evoke voiding contractions [34]. Urodynamic data (voiding and nonvoiding events, voided volumes, and animal movements or spasms) and experimenter notes were recorded on video for offline playback and analysis with Datawave software (http://www.dwavetech.com/). Voiding efficiency was calculated as the percent volume voided per volume saline infused. Cystometrogram (CMG) parameters are the mean of 5 consecutive cycles (which were sampled approximately 15 minutes after the start of saline infusion). CMGs were analyzed for resting pressure (mm Hg), maximal amplitude of contraction (mm Hg; peak pressure minus resting pressure), contraction time (sec), and intercontraction interval (sec).

*Activity Meter*. The in-cage activity of every animal was recorded using an Opto-M3 infrared activity monitor (Columbus Instruments, Columbus, OH). A cradle equipped with infrared beams, spaced one inch apart, was placed around the cage so that the infrared beams shined across the cage near the floor. The data were collected as ambulatory (number of two consecutive beam breaks; i.e., the animal was moving across the cage) and total movements (number of beam breaks) during the active phase (6 pm–6 am).

### 2.3. Histology of Lesion Epicenter

Each animal was deeply anesthetized with ketamine/xylazine and perfused transcardially with a solution of normal saline and heparin. The bladder was blunt dissected away from the prostate, blotted dry, and weighed. The spinal lesion area was removed and placed in 4% paraformaldehyde for at least 48 hours, followed by 30% sucrose/phosphate buffer solution with 1% sodium azide for at least 24 hours and until the tissue was cut on a cryostat (Leica CM 1850) at 18 *μ*m thickness and stained with both Luxol fast blue and cresyl violet (Kluver-Barrera method). The lesion area was quantified as previously described [30] using Spot Advanced software (Diagnostic Instruments, Sterling Heights, MI) and the Nikon E400 microscope. Briefly, white matter was divided into four regions (dorsal columns, dorsolateral funiculus, ventrolateral funiculus, and the ventral funiculus) and each area was subdivided into left and right sides. The gray matter was divided into dorsal and ventral regions. The central canal, medial edges of the dorsal horn, and the tips of the ventral horn were used as landmarks for the divisions. The percent of white matter sparing (WMS) was determined by dividing the white matter remaining at the epicenter, .5 mm rostrally, and 1.0 mm rostrally by the average area of white matter present in intact sections. The intact area of white matter for a given region was estimated by averaging together measurements from 2 sections 2 mm rostral to the epicenter.

### 2.4. Statistics

SCI animals were excluded from analysis if the recorded displacement of the impactor tip was less than 1.0 mm during the injury (*n* = 3); these animals typically have a very mild injury. An additional 2 animals were sacrificed during the first two weeks following injury due to autophagia. This resulted in the following groups: train, *n* = 13; nontrain, *n* = 7; forelimb, *n* = 9; sham, *n* = 4. Analyses were performed using SigmaStat and Microsoft Excel. Levene's test for inequality of variance was performed. One-way repeated measures analysis of variance (ANOVA) (fixed effects) was performed for tests of within subject and between subject effects followed by Bonferroni* post hoc t*-tests for the BBB, allodynia, and activity. One-way ANOVA (fixed effects) was used for cystometry parameters, bladder weight, gait, and kinematics followed by Bonferroni* post hoc t*-tests, significance level *p* < .05. For the regularity index (RI),* post hoc* tests approached significance and were followed by the Mann-Whitney *U* test. Percent animals with consistent weight support were analyzed with the binomial proportion test. Significance level was *p* < .05. All values reported in the paper are mean ± SD.

## 3. Results

### 3.1. Training Significantly Improved Overground Locomotion

**Figure 1:**
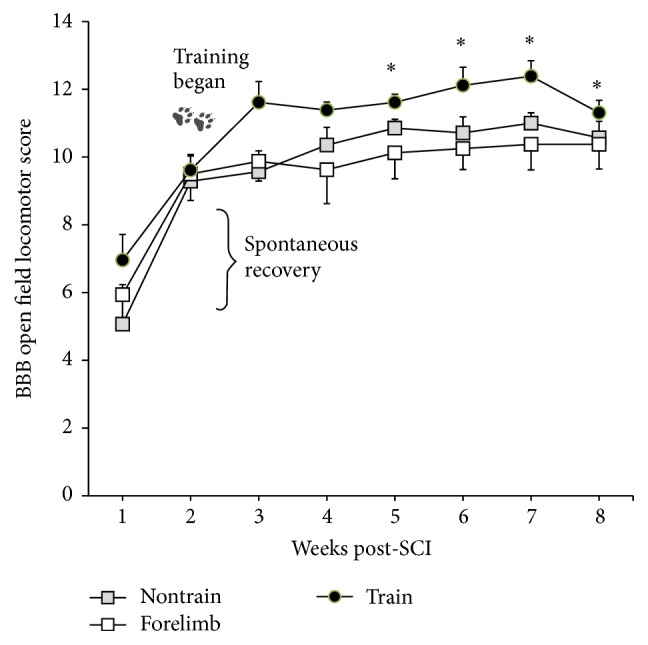
Weekly open field locomotor scoring of trained, nontrained, and forelimb SCI male rats. Statistically significant locomotor recovery occurred from week 1 to week 2 for all groups. At 2 weeks post-SCI training began. Only the trained group had significant increases from pretraining. Shams not shown, BBB = 21 (*∗* versus pretraining; W2; repeated ANOVA with Bonferroni* post hoc t*-tests: nontrain *n* = 7; forelimb *n* = 9; train *n* = 13).

**Figure 2:**
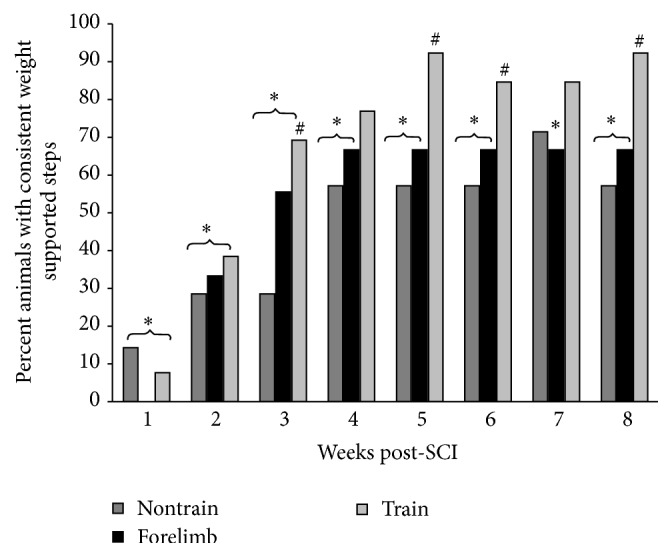
Percent animals with consistent weight support. Significant differences were found between sham and both the nontrained and forelimb groups (*∗*) at weeks 1–8, except week 7, as well as between the trained and nontrained groups (#) at weeks 3, 5, 6, and 8. No differences were found between sham and trained animals after week 3 (binomial proportion test: nontrain *n* = 7; forelimb *n* = 9; train *n* = 13).

All groups demonstrated significant spontaneous recovery from 1 to 2 weeks after SCI. At the initiation of training (2 weeks after SCI) all groups functionally displayed plantar paw placement with occasional weight support (BBB scores: trained: 9.61 ± 1.7; forelimb: 9.5 ± 2.2; nontrained 9.3 ± 2.0). After 3 weeks of training (5 weeks after SCI), trained animals' BBB scores were significantly higher compared to pretraining and remained significantly higher through the rest of the study (Figure 1). Forelimb and nontrain controls did not significantly improve from their pretraining BBB score. Consistent weight supported stepping (BBB ≥ 11) is a functional milestone on the BBB scale (which makes each animal eligible to receive a subscore). A significantly higher proportion of animals in the trained group achieved consistent weight support. No differences were found between sham and trained animals after week 3 (Figure 2). However, the trained group was also not significantly different from the forelimb or nontrain groups. Forelimb and nontrain groups had significant proportions of animals* unable* to consistently weight-support as compared to sham throughout the study.

**Figure 3:**
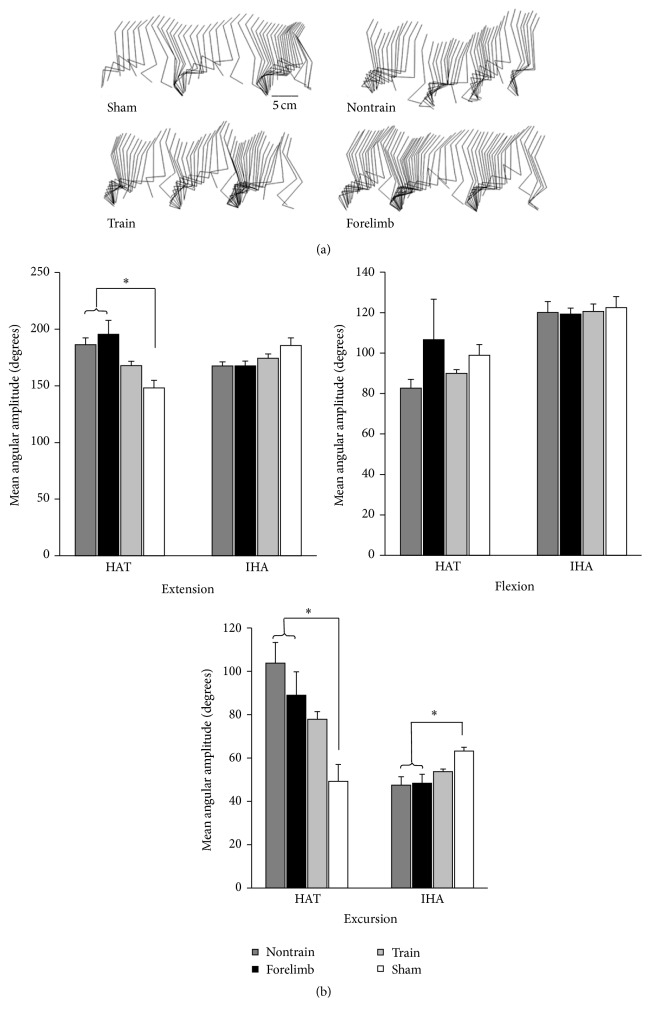
(a) Kinematic illustration of the hindlimb (iliac crest, greater trochanter, knee, lateral malleolus, and metatarsophalangeal joint) during stepping. (b) The maximum (extension) and minimum (flexion) angles as well as excursion (range of motion) of the hip-ankle-toe and iliac crest-hip-ankle angles were calculated and compared between trained (*n* = 13), nontrained (*n* = 7), forelimb (*n* = 9), and sham (*n* = 4) (ANOVA with Bonferroni* post hoc t*-tests). Significant differences were found for ankle extension and hip and ankle excursion (*∗* sham versus nontrained and forelimb). Sham was not different from trained.

Kinematic measurements of overground locomotion revealed that quadrupedal-trained animals more closely approximated the hindlimb angular movement of shams (no significant differences). However, the trained group was also not significantly different from the forelimb or nontrain groups. Both nontrained and forelimb trained control groups had significantly larger angular excursions of the hip and ankle, as well as significantly larger ankle extension (Figure 3).

**Figure 4:**
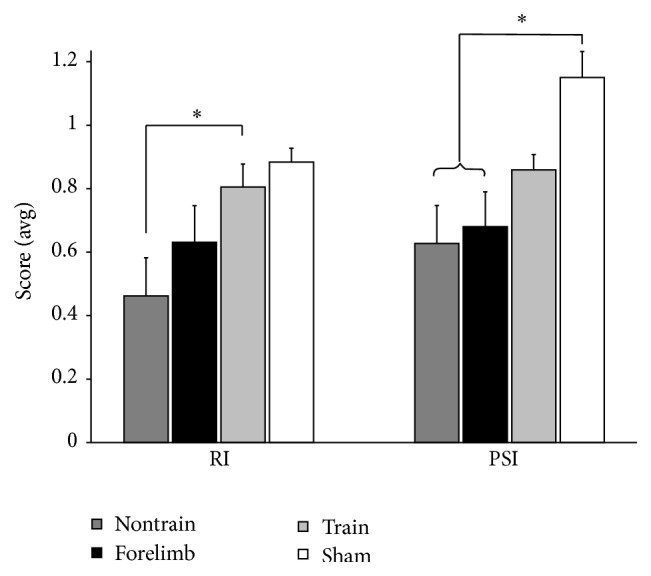
The regularity index revealed a significant difference between the trained and nontrained groups (*∗*) (Mann-Whitney *U* test). The plantar stepping index revealed a significant difference between the sham and both the nontrained and forelimb groups (*∗*) (ANOVA with Bonferroni* post hoc t*-tests). Parameters of gait were not significant: stride length, stride time, base of support, and toe velocity. Nontrain *n* = 5; train *n* = 13; forelimb *n* = 6; sham *n* = 4.

The trained animals recovered to BBB scores necessitating the assessment of forelimb-hindlimb coordination. Importantly, although the BBB can be used to assess coordination, we also utilized a more objective assessment of coordination, the regularity index (RI) [35]. The RI is a score of plantar footfall pattern and limb coordination [36], and the trained group scored significantly better than the nontrained group (Figure 4). The plantar stepping index (PSI), which is a ratio of plantar hindlimb to forelimb steps [27], revealed no differences between trained and sham controls, while nontrained and forelimb controls scored significantly lower than shams (Figure 4). However, the trained group PSI was also not significantly different from the forelimb or nontrain groups. Analyses of gait parameters did not detect differences between groups for stride length, stride time, base of support, or toe velocity (data not shown).

The horizontal ladder demonstrated a large difference between injured and sham animals. Sham animals were able to cross the horizontal ladder with only one or two errors (1.1 ± 0.51). Although trained animals displayed improvements in overground locomotion and limb coordination, their ability to control fine placement of the hind paw remained impaired. There were no differences between any SCI group (train 7.67 ± 2.88; forelimb 8.43 ± 2.44; nontrain 7.42 ± 3.80).

### 3.2. At-Level Allodynia

**Figure 5:**
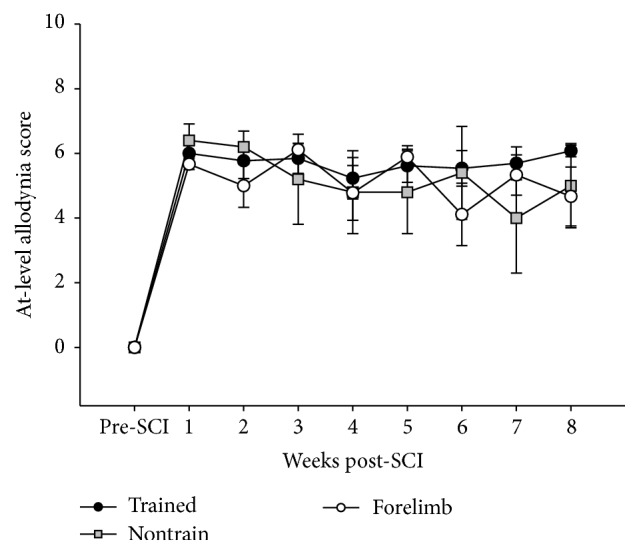
No animals were sensitive to touch or gentle squeeze prior to injury. Immediately after SCI, all rats exhibited a moderate degree of evoked at-level allodynia. There were no differences between groups regarding the course of at-level allodynia.

Quantitative measurements of sensitivity to mechanical stimuli were obtained on two separate occasions prior to injury and then once per week for 8 weeks after SCI in the trained, nontrained, and forelimb groups. Preinjury measurements revealed that none of the groups demonstrated allodynic behavior to innocuous stimuli and were equivalent at baseline. One sham animal vocalized prior to injury and was excluded from this analysis only. The average sham score after surgery was 1.75 ± 2.4. Immediately after SCI, all rats exhibited a moderate degree of evoked at-level allodynia consisting of either freezing, escaping, or grabbing (with or without vocalization) towards a stimulus filament ranging from 15 g to 0.008 g. These pain-related aversion behaviors persisted throughout the course of training for all SCI groups (allodynia score at 8 weeks after SCI: train 5.72 ± 1.44; forelimb 5.19 ± 1.47; nontrain 5.23 ± 2.33) (Figure 5).

### 3.3. Cystometry and Bladder Weights

Autonomic dysfunction is of high priority for individuals with SCI [37]. We examined a total of 12 urodynamic parameters as well as bladder weights to identify possible treatment effects. Only a few are reported here. Shams were directly compared to SCI groups for bladder weight but not for urodynamics, as the fill rate of .25 mL/min causes different physiological outcomes in a very small bladder compared to a bladder twice the normal size. No significant differences were observed between trained, nontrained, and forelimb groups (one-way ANOVA, *p* > .05). Bladder weights of all SCI animals were larger than shams (bladder weight in milligrams: sham 143.3 ± 25.2; train 226.5 ± 52.7; forelimb 287.2 ± 78.9; nontrain 240.0 ± 65.6; one-way ANOVA *p* = .013) [38, 39]. Intercontraction interval (ICI, seconds) was not different between groups (train 130.5 ± 88.8; nontrain 120.0 ± 51.1; forelimb 127.2 ± 77.4). Voiding efficiency was not different between groups (train 89.5 ± 9.8; nontrain 92.3 ± 8.3; forelimb 86.1 ± 15.2).

### 3.4. Home Cage Activity (6 pm–6 am)

**Figure 6:**
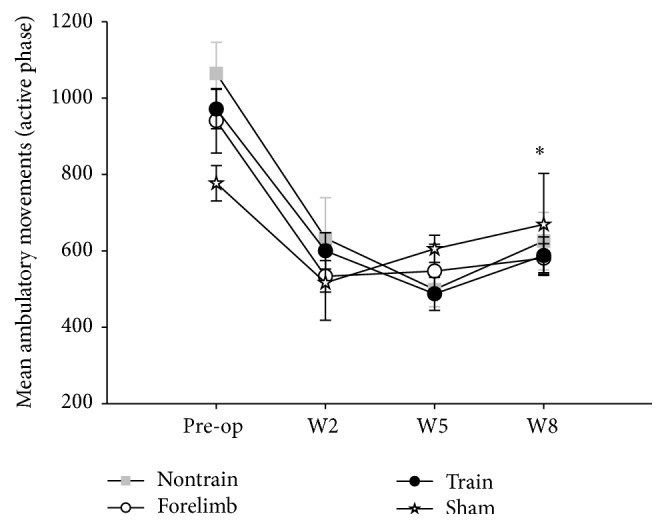
Home cage activity: in-cage activity assessments with an infrared activity monitor show no differences in the amount of ambulatory movements up to week 8 (W8), suggesting that the increased locomotor recovery of the trained group was a result of the step training paradigm and not due to “self-training.” All animals significantly decreased activity with time from surgery (*∗* preoperation versus W8 for each group; repeated ANOVA with Bonferroni* post hoc t*-tests; nontrain *n* = 7; train *n* = 13; forelimb *n* = 9; sham *n* = 4).

Another source of potential locomotor practice is in-cage activity. Home cage activity was monitored during the active phase and revealed that while all groups significantly decreased activity with time, there were no significant differences between any group when measuring ambulatory or total in-cage movements. All groups followed a similar pattern (Figure 6). This finding suggests that home cage activity was not a significant contributor to improved locomotion in the trained SCI group.

### 3.5. White and Gray Matter Spared

**Figure 7:**
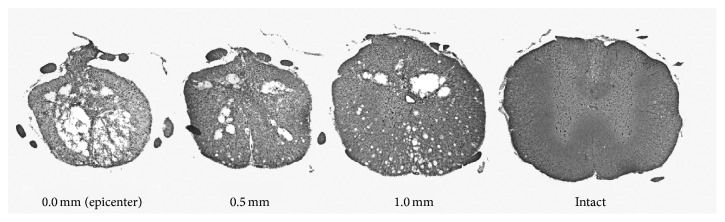
Representative spinal cord segments. Histological assessment did not reveal any differences between groups when analyzing total white or gray matter from the epicenter to 1.0 mm rostrally or when further subdividing the sections into areas of ventral and ventral lateral funiculi (ANOVA: nontrain *n* = 7; train *n* = 13; forelimb *n* = 9).

Histological assessment of the injury started with the epicenter and continued 1 mm rostrally.  Figure 7 shows representative sections of the contused cord at the epicenter and .5 mm and 1.0 mm rostrally from the injury as well as an intact section. There were no significant differences between any group when analyzing weight gain (each group gained approximately 100 grams), injury parameters, white matter at the epicenter, .5 mm, or 1.0 mm (Table 1). The white matter was further subdivided into ventral and ventral lateral funiculi and gray matter of the ventral horn was also calculated at the epicenter, .5 mm, and 1.0 mm. There were no differences detected between any group when analyzing the ventral portions of the spinal cord. These data suggest that functional differences observed between groups cannot be attributed to differences in the amount of spared white or gray matter. Yet, plasticity within the spared pathways may facilitate functional recovery.

## 4. Discussion

The method of training used in this study facilitated additional recovery of the trained group (beyond the substantial amount of spontaneous recovery that occurred during the first two weeks following SCI [18, 26, 40]) and is the first to show overground improvements using quantitative kinematic and gait analyses in addition to qualitative open field scoring for contused male rats. The differences in locomotor parameters of trained versus nontrained and forelimb controls could not be attributed to differences in home cage activity, similar to a study comparing spontaneous exercise and enriched environment in which the amount of activity did not correlate to locomotor recovery [41]. Additionally, like other studies using activity based therapies [16, 18, 42], we found that training did not increase white matter sparing, even when analyzing subdivisions of the spinal cord, such as the ventrolateral funiculus. These results suggest that training reinforced or facilitated plasticity within the remaining pathways or lumbosacral circuits rather than promoting regeneration or sprouting of new pathways.

After 6 weeks of training, almost all trained SCI animals had achieved weight supported stepping and degrees of forelimb-hindlimb coordination. In contrast, while many animals in the SCI control groups recovered consistent weight supported stepping, recovery was slower and some animals never regained the ability to weight-support. These data are consistent with stand training in transected cats, which increased the duration of hindlimb standing [43, 44]. In rats with contusion or compression injury, treadmill training improved ankle extension [14] and weight bearing during open field locomotion [16]. Compared to our previous findings [20], as little as 30 minutes of daily locomotor training can improve weight bearing locomotor ability in SCI animals (60 minutes of daily training did not further improve BBB scores).

Many studies perform quantitative kinematic measurements on the treadmill (bipedal and quadrupedal) where the hindlimb is passively extended during stance, the trunk is supported by a harness, and partial body weight support is provided. Here, we used overground kinematic and gait analyses to quantify the training effects on full weight bearing overground locomotion compared to sham and SCI controls. Ankle extension and ankle and hip excursion were normalized by training (more similar to shams). Coordination and plantar paw placement were also normalized (RI and PSI). In cats, treadmill training increases the recruitment of flexor motor pools compared to nontrained cats [6]. Trained SCI cats and rats have also been shown to have greater paw lift and hip flexion, respectively, both allowing a reduction in paw dragging [6, 15, 45]. Our results extend these studies and indicate that training facilitates normal step cycle trajectories during* overground* locomotion by decreasing ankle extension and excursion, increasing hip excursion, and increasing the plantar stepping index.

Step training that provides alternate limb loading and rhythmic repeated steps can promote neural activity and improve EMG patterns and amplitudes [17, 46, 47], which could explain how ankle extension (during stance), plantar paw placement, and weight support improved with training. Importantly, not all aspects of motor control improved with quadrupedal training. Although training promoted the recovery of overground locomotion, training did not improve the ability to cross a horizontal ladder. The ladder test correlates very well with injury severity [48]. However, this task requires precision paw placement and relies on proprioception [49, 50]. While our training paradigm improved gross locomotion (weight bearing, coordination, etc.), fine proprioceptive movements remained impaired, possibly due to the task specificity of training.

Locomotor training has been reported as having beneficial effects on bladder function in clinical settings [51–53]. Indeed, we previously demonstrated that 60 min of training significantly improved bladder function. However, our results according to cystometry and bladder weights in this study and SCI-induced polyuria in our previous study [54] indicate that 30 minutes of training did not affect bladder function after SCI. Thus, longer training durations should be further investigated for nonlocomotor functional recovery. Indeed, we found that, with 60 minutes of training, both quadrupedal and forelimb training improved the maximum bladder contraction amplitude and reduced SCI-induced polyuria [55].

With respect to sensory function, the emergence of neuropathic pain after SCI significantly impacts patients' quality of life and interferes with functional recovery [56–58]. In the clinical setting, exercise, including treadmill training, can positively influence neuropathic pain [59–61]. We previously demonstrated that 60 min of locomotor training resulted in a significant improvement in at-level allodynia scores compared to the nontrained group. This effect was observed after 3 weeks of training and remained consistent throughout the course of training [20]. Other rodent models of SCI, employing varying degrees of locomotor training intensity and duration, also report attenuations of allodynic responses following either below-level to the plantar aspects of the paws [62, 63] or at-level [64] mechanical threshold testing. In this study, 30 minutes of step training was not sufficient to ameliorate the onset of trunk at-level allodynia after SCI. Differences across studies may be attributed to the spinal location (cervical versus thoracic) and type of injury model (contusion versus compression), gender, magnitude, and intensity of training as well as the region being tested (trunk versus paw). It is not clear whether step training induces a level of resistance to the glabrous plantar aspect of the paw, perhaps influencing mechanical withdrawal thresholds. Overall, the degree of spared spinal pathways, spontaneous recovery, and the rhythmic weight bearing load during stepping, which may promote activation of cutaneous and proprioceptive afferents, may all be driving factors influencing improvements in tactile sensation [63]. A comparison between males and females may also be warranted as the development of pain and the requirements for differential effective exercise protocols may be sex-dependent [65–67].

Enriched environments and spontaneous exercise, such as wheel running, have been shown to improve locomotor recovery after SCI [41, 42, 68]. The possibility exists that exercise could promote post-SCI recovery through more general mechanisms, such as through alterations in inflammatory pathways, a “nursing effect” through neurotrophins, or increased well-being. In this study, we utilized a control group to induce exercise without specific activation of lumbar circuitry. This forelimb trained SCI control group did not show any benefits of exercise. In fact, although not statistically different, the forelimb trained SCI animals scored slightly worse on multiple locomotor parameters, including BBB score, ankle flexion and extension, and ladder errors. These findings suggest that step training improves locomotor recovery through direct activation of lumbar circuitry. In contrast, according to our finding that 60 minutes of forelimb training significantly altered some parameters of urinary tract function [55], other mechanisms must be responsible for these nonlocomotor improvements. In conclusion, a number of factors can enhance or prevent functional recovery after SCI. These factors are related not only to the injury but specific parameters of step training. These factors have been widely varied in experimental SCI and are likely contributing to different conclusions about step training's efficacy in an incomplete rat model of SCI. Our results suggest that 30 minutes of manually assisted step training initiated in the subacute stage of recovery can maximize potential locomotor gains. We found that 30 minutes of quadrupedal step training improved overground weight support, coordination, and ankle/hip range of motion. These improvements could not be directly attributed to lesion variability or home cage activity. We also found that 30 minutes of daily training did not improve precision paw placement, bladder function, or allodynia. While 60 minutes of daily training does not result in even higher BBB scores or an increase in the percentage of animals that can weight-support, longer daily training sessions result in additional benefits to nonlocomotor functions (allodynia and bladder function), which would substantially influence a patient's quality of life.

## Figures and Tables

**Table 1:** 

Treatment	Force (kdyn)	Displacement (*μ*m)	WMS% 0.0 mm	WMS% 0.5 mm	WMS% 1.0 mm	Body weight at week 8 (g)
Nontrain	215.57 ± 5.6	1269.4 ± 117.2	9.2 ± 5.1	28.3 ± 8.2	65.0 ± 5.7	417.57 ± 43.8
Forelimb	217.13 ± 4.6	1308.8 ± 140.0	11.4 ± 8.6	27.6 ± 9.0	59.4 ± 11.1	439.7 ± 43.1
Train	217.45 ± 6.6	1269.6 ± 158.9	12.8 ± 7.1	26.9 ± 8.8	59.6 ± 12.7	441.2 ± 35.0
